# Association of immune cell recruitment and BPD development

**DOI:** 10.1186/s40348-022-00148-w

**Published:** 2022-08-02

**Authors:** Motaharehsadat Heydarian, Christian Schulz, Tobias Stoeger, Anne Hilgendorff

**Affiliations:** 1 Institute for Lung Health and Immunity and Comprehensive Pneumology Center with the CPC-M bioArchive, Helmholtz Center Munich, Member of the German Center for Lung Research (DZL), Munich, Germany; 2 German Center for Cardiovascular Research (DZHK), Partner Site Munich Heart Alliance, Munich, Germany; 3 Department of Medicine I, University Hospital, Ludwig Maximilian University, Munich, Germany; 4 Center for Comprehensive Developmental Care (CDeCLMU) at the interdisciplinary Social Pediatric Center, (iSPZ), University Hospital Ludwig-Maximilian University, Munich, Germany

**Keywords:** Neonate, Lung, Chronic lung disease, Bronchopulmonary dysplasia, Inflammation, Neutrophil, Monocyte, Macrophage

## Abstract

In the neonatal lung, exposure to both prenatal and early postnatal risk factors converge into the development of injury and ultimately chronic disease, also known as bronchopulmonary dysplasia (BPD). The focus of many studies has been the characteristic inflammatory responses provoked by these exposures. Here, we review the relationship between immaturity and prenatal conditions, as well as postnatal exposure to mechanical ventilation and oxygen toxicity, with the imbalance of pro- and anti-inflammatory regulatory networks. In these conditions, cytokine release, protease activity, and sustained presence of innate immune cells in the lung result in pathologic processes contributing to lung injury. We highlight the recruitment and function of myeloid innate immune cells, in particular, neutrophils and monocyte/macrophages in the BPD lung in human patients and animal models. We also discuss dissimilarities between the infant and adult immune system as a basis for the development of novel therapeutic strategies.

## Background

Bronchopulmonary dysplasia (BPD) is the neonatal form of chronic lung disease occurring in the context of prematurity and is characterized by impaired pulmonary development [1]. The consequences of misdirected lung development persist into adulthood, and although the advances in neonatal intensive care decreased the rate of overall mortality after premature birth, the prevalence of chronic complications like BPD remained [2, 3]. Different prenatal and postnatal factors have been introduced as contributors to BPD development, including genetic/epigenetic risk factors, intrauterine hypoxia and growth retardation, infection, mechanical ventilation (MV), and oxygen supplementation [4, 5]. Clinically, BPD is classified into three severity grades according to the need for oxygen supplementation or MV 28 days after birth and near term age, i.e., 36 weeks gestational age (GA) [6]. More than half of the preterm infants that require life-saving postnatal treatment with MV or oxygen supplementation develop subsequent complications such as failure of alveolo- and angiogenesis [7, 8]. Premature babies born before 32 weeks of GA present with a structural and functional immaturity of the lung that includes an immune system not yet equipped to sufficiently respond to environmental insults. The subsequent vulnerability to infections and injury together with the misdirected role of immune functions in organ development highlights the importance of better understanding—and potentially targeting—immune-related phenomena [9]. Inflammation is a vital element of host defense [10]. Excessive or persistent inflammation, however, is known to interfere with organ development including the lung, and thus, a key contributor to the emergence and pathogenesis of BPD indicating disease progression [11, 12]. Inflammation can be triggered by both prenatal as well as postnatal factors including hyperoxia, MV, and infections [13–16] that act beyond the background, e.g., immaturity and gender [16]. Preterm labor per se is associated with intrauterine infections with ureaplasma and mycoplasma infections holding a specific role in BPD development [13]. In consequence, intrauterine and early postnatal infections and the respective inflammation are common in premature infants [17].. The immune system in premature infants is still undeveloped with a lower number of neutrophils and monocytes in the cord blood, resulting in a greater vulnerability to infections and a significant imbalance of pro-and anti-inflammatory mechanisms upon injury [18, 19].

The later development of BPD is then characterized by the accumulation and activation of myeloid leukocytes cells in the lung, which in turn drive pathophysiological processes such as an enhanced permeability of the endothelial and epithelial barrier (“leakage”) [20, 21]. Recently, studies specifically focused on the impact of the accumulation of inflammatory cells, myeloid neutrophils, and monocytes in particular, on misguided alveolar and pulmonary vascular development as the hallmark of BPD [22–24] However, the bidirectional role of resident and recruited myeloid cells in development and injury has been targeted for the development of treatment strategies. This review provides an overview of the current state of knowledge available on the known and potential role of neutrophils, monocyte/macrophages, and their crosstalk with resident cells in BPD development, side by side with a discussion about the main challenges concerning modeling the disease and future perspectives in the field. The important differences in immune functions when compared to adults are highlighted.

### Innate immunity in the development of neonatal chronic lung disease

Due to the limited exposure to foreign antigens in utero*,* the newborn infant relies on innate immunity-dependent defense strategies as the adaptive immune response is still naïve [25–27]. Also, the shortened time for the prenatal development of immune functions in preterm infants impacts immune responses immediately after birth [28]. Circulating and resident myeloid immune cells such as neutrophils and monocytes are at the forefront of the innate immune response and act as potential triggers of inflammatory signals including cytokines and chemokines [29], which mediate immune activation as well as the transition from innate to adaptive immunity [30, 31].

**Fig. 1:**
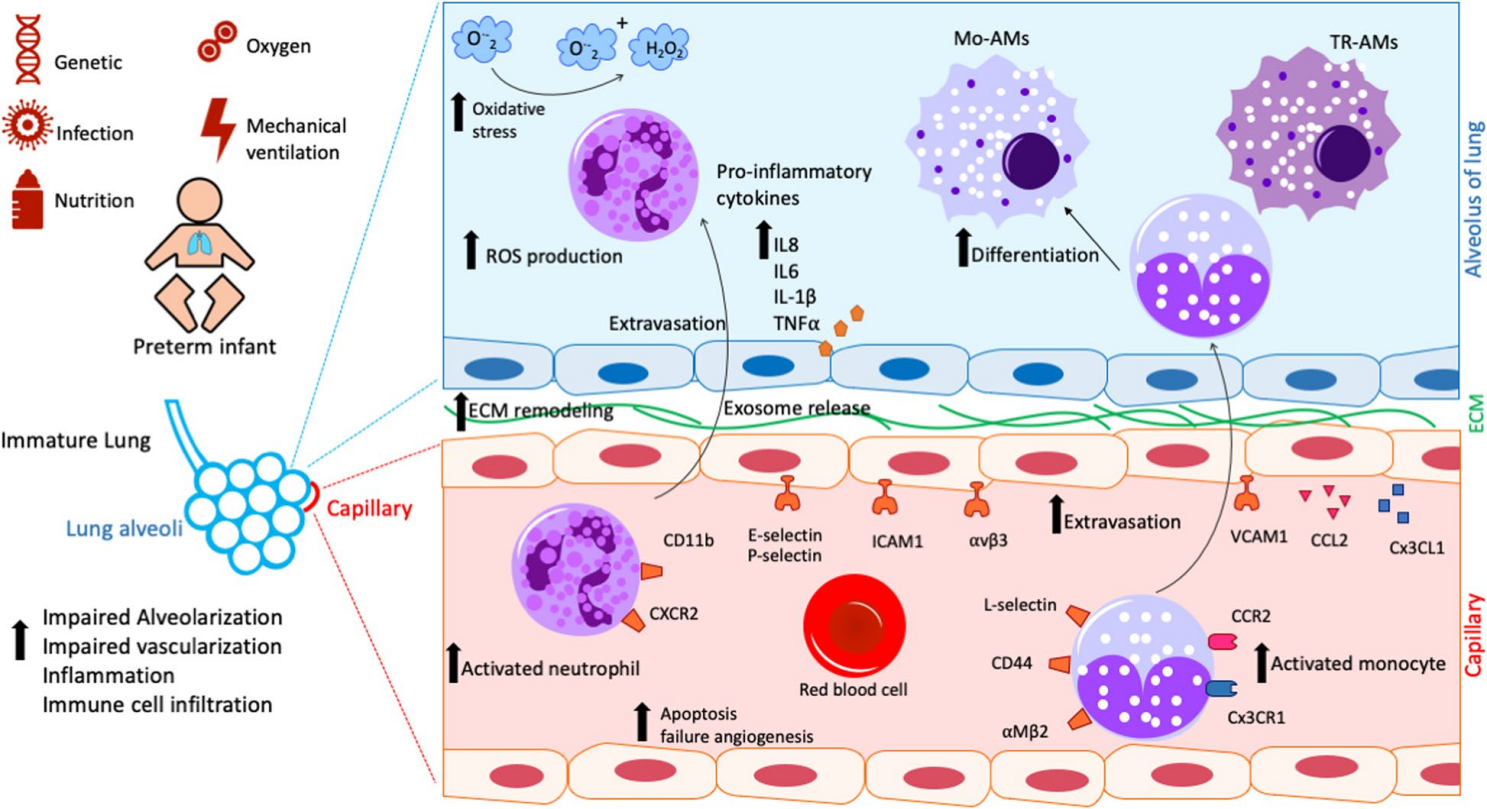
Schematic represents the innate immune signals related to lung inflammation culminating in the BPD development and progression. Preterm infants suffer from BPD due to the impact of various risk factors including genetic background, prenatal and postnatal infections, nutrition, oxygen toxicity, and mechanical ventilation. Exposure of the structurally and functionally immature lung to these risk factors provokes oxidative stress and results in the increased expression of pro-inflammatory cytokines by resident cells in the alveolar niche. Subsequently, innate immune cells are recruited including neutrophils as the first-line defense. These events are followed by the extravasation of monocytes which eventually differentiate into macrophages in the tissue context. Neutrophil and monocyte signaling is associated with pulmonary tissue damage including impairment of epithelial and vascular function and progression of inflammatory processes. Black arrows indicate the elevating events during BPD

Environmental assaults not only encompass bacterial or viral infections but exposure to toxins and mechanical stress as initiated by MV and oxygen supplementation all triggering inflammation involving both innate and adaptive immune responses [32, 33] (see Fig. 1). The airway epithelium is an immunologically active barrier and the main source of pro-inflammatory cytokines [34, 35]. Upon the airway epithelial injury and local release of the classical pro-inflammatory cytokines of the innate immune defense such as interleukin-1, -6, -8 (IL-1, IL-6, IL-8), and tumor necrosis factor-alpha (TNFα), blood neutrophils immediately migrate into the lung tissue, subsequently followed by monocytes which—once recruited—rapidly differentiate into macrophages [36–38]. This monocyte-to-macrophage differentiation is initiated after the emigration from the circulation into the tissue in association with gaining different functional phenotypes dependent on the local tissue environment [39]. Depending on the micro-environmental signals, macrophages develop distinct functions represented in the concept of their differentiation into classical activated, inflammatory (M1) or activated, anti-inflammatory, or fibrotic (M2) states [38]. Furthermore, neonatal and adult alveolar macrophages (AMs) differ from each other and present their unique transcriptome profile under the impact of micro-environmental signals [40].

Studies on BPD research during the last few decades primarily focused on the association between cytokine and chemokine expression patterns and disease onset or progress [41]. Researchers found high expression levels of specific cytokines stemming from classical macrophage activation such as monocyte chemoattractant protein-1 (MCP-1/CCL2), macrophage inflammatory proteins (MIP), the neutrophil chemokine IL-8, and low expression of IL-10 in association with BPD development [12, 42–45] while expression other cytokines, for example, IL-4 and IL-13 did not correlate with BPD [46]. In addition to the myeloid lineage, an increase in type 3 innate lymphoid cells (ILC3) have been reported for their pro-inflammatory role in BPD development, mainly through the secretion of IL-17, which is known as a key factor in the recruitment of neutrophils via stimulation of IL-8 including neutrophils and CXCL1 and CXCL2 chemokines [47, 48]. In contrast, levels of granulocyte colony-stimulating factor (G-CSF), responsible for the stimulation of granulocyte production in the bone marrow (BM), were found to be reduced in preterm when compared to term infants, which indicates the defective function of neutrophils during infection [49]. Thus, a potential therapeutic approach to treat or prevent BPD has been proposed by inhibiting the unsolicited expression of cytokine and chemokine [50, 51], which plays a critical role in preterm infants innate immunity and BPD development.

*To delineate specific cellular functions engaged in the innate immune response, we, in the following, specifically outline neutrophil and monocyte/macrophage functions and interactions in light of the sequential order of immune events.*

### Neonatal neutrophils and their activation in BPD

Neutrophils play an indispensable role in acute lung inflammation in both mature and developing organisms [52–54]. During the initiation phase of inflammation, neutrophils undergo a variety of changes in gene expression and functional properties [55]. Neutrophilic granulocytes originate in the BM and are released into circulation [56], where they act as the first line of cellular immune defense when getting recruited to the site of injury [57]. This first wave of inflammation is followed by the recruitment of monocytes in a later stage. Neutrophils eliminate pathogens through phagocytosis and via releasing proteases, reactive oxygen species (ROS), and bioactive membrane vesicles through a function called degranulation [58]. ROS are highly reactive species formed from O_2_, such as hydroperoxyl and hydrogen peroxide, and can be transformed into radicals [57, 59]. The sudden postnatal exposure of the immature lung to oxygen and MV is currently seen as the inducer of ROS-dependent local and systemic neutrophilic inflammatory responses [60–62], that in turn can cause the release and activation of neutrophils and increased ROS production [63, 64]. Next to the ROS-induced effects that restrict lung development and contribute to BPD, another destructive role of activated neutrophils in the process of alveolar formation is likely related to the release of exosomes that can increase the proliferation of airway smooth muscle cells, induce remodeling and extracellular matrix (ECM) destruction—all being features of BPD [65–68]. In addition, the release of neutrophil elastase (NE) and metalloproteinases (MMP) like MMP-9 can cleave fibronectin and increase ECM degradation in the alveoli, thereby contributing to scaffold remodeling and BPD progress [69, 70]. Alternatively, these factors can be released by the ECM itself, thereby perpetuating the vicious cycle of damaging the alveolar niche [68]. In line with this, endogenous protease inhibitors, such as serine protease inhibitor (SERPIN)B1 have been demonstrated to hold protective functions against neutrophil protease-induced tissue damage in BPD [71].

In comparison to adult immune functions, term and preterm newborn infants show fewer peripheral blood neutrophil counts [72]. At 22–23 weeks of gestation, only 2% of all leukocytes in the peripheral blood are neutrophils, while these counts increase to 60% in term-born infants [55] and about 60–70% in adults [73]. Functionally, neonatal neutrophils show the deficiency to form neutrophil extracellular traps (NETs) [74] as well as a lower cell surface expression of L-selectin [72]. Following injury, endothelial cells express leucocyte adhesion molecules on their luminal side, particularly P- and E-selectins, together with several integrin members of the ICAM family, to allow adhesion of neutrophils through their respective selectin ligands and integrins, e.g., CD18 and CD11b [75]. Neonatal neutrophils, however, express lower levels of CD11b and subsequently display only attenuated abilities for adhesion as compared to adult cells. Also, reduced neutrophil CD18 and L-Selectin expression levels have even been described for their predictive value in BPD [76] while it has been reported that the ratio of blood neutrophil to lymphocyte has been increased 72 h after birth [12]. The latter points to an inflammatory stimulation in utero encompassing the lung. Nevertheless, reduced blood neutrophil levels have to be viewed in light of a potential imbalance of tissue accumulation and BM production [77]. The vulnerability of the newborn lung towards inflammatory injury is highlighted by findings in bronchoalveolar lavage fluid (BALF) demonstrating the combination of MV, hyperoxia, and inflammation to trigger the expression of acute-phase cytokines and chemokines in newborn infants [78] together with increased neutrophil apoptosis where intact neutrophils are phagocytosed by AMs before degranulation [79]. These findings might reflect a mechanism contributing to sustained inflammation and tissue damage. Neutrophil-related events as a therapeutic approach are still being explored.

*The second wave of inflammation, induced by the neutrophil through releasing chemoattractants, involves the recruitment of other immune cells, specifically monocytes/macrophages.*

### Monocytes/macrophages and their response in BPD developing infants

Since the lung is the interface to the external environment and related immunological challenges [80], mononuclear phagocytic cells are dedicated to confronting these challenges. Therefore, in response to environmental insults, circulating monocytes migrate into the alveolar space and contribute to the process of alveolar injury and remodeling [81, 82]. Monocyte/monocyte-derived alveolar macrophages (Mo-AMs) recruitment to the lung is a characteristic process in BPD [83], even in light of a reduced number of monocytes in the neonatal peripheral tissue per se [84] and their lower adherence and transmigration capacity when facing the non-inflamed endothelium [84].

The process seems to mark chronification in BPD despite the potential to contribute to the resolution of inflammation in the first place. Like neutrophils, monocytes originate from the granulocyte monocyte progenitor (GMP) in the BM and play a key role in innate immune responses which are closely related to vascular homeostasis [85, 86] and contribute to processes such as phagocytosis and lung regeneration at the same time. These functions have in part been attributed to modulating alveolar stem cells [87, 88].

Current concepts relate to three human monocyte subsets: classical (CD14^+^CD16^−^), non-classical (CD14^-^CD16^+^), and intermediate (CD14^+^CD16^+^) monocytes [89]. Each subset holds unique functions represented by different surface markers [90]. The differing abilities regarding recruitment, cytokine production, and capability of endothelial activation [91] relate to the grouping introduced above. Whereas nonclassical/intermediate monocytes secrete higher levels of TNFα and demonstrate an increased capacity for endothelial transmigration, classical monocytes secrete high levels of IL-6, and assist neutrophil recruitment [91]. In mice—in relation to the human concept—main types of monocytes are identified according to three subsets including classical (Ly6C^++^, CCR2^++^, CD43^+^), nonclassical (Ly6C^+^, CCR2^+^, CD43^++^, CX3CR1^+^), and intermediate (Ly6C^++^,Treml4^+^, CD43^++^) monocytes [92–95]. Here, Ly6C+ nonclassical monocytes continuously scan the endothelium for injury and infections [96] and are thereby involved in maintaining endothelial barrier integrity, regeneration, and repair [97–99].

Monocytes respond to chemokine signals with the adhesion to endothelial cells following a multistep adhesion cascade involving the interaction of leukocyte adhesion molecules for instance L-selectin, PSGL1, LFA1, MAC1, VLA4, and their respective receptors on endothelial cells from the selectin and immunoglobulin superfamily [85, 86, 100–102]. Adhesion dynamics and homeostatic extravasation is depending on CD31 and CD54 expression levels [84]. Monocytes from preterm infants, however, show a lower surface expression of the CD11b/CD18 and CD31 adhesion receptors, resulting in a reduced ability for extravasation and greater susceptibility to infections [84, 103]. Necessary for the subsequent differentiation of fetal monocytes into pre-AMs during embryonic development, the transforming growth factor (TGF)-β1 is simultaneously a key player in lung development. Its dual role next in inflammation and apoptosis [104, 105] and the dynamic regulation during alveolarization renders the prenatal regulation of TGF-β during in utero inflammation an interesting target [106, 107]. TGF-β1 is an important factor for AM maturation after birth as well as for the homeostasis of adult AMs [108] and provides important signals for monocyte recruitment [109] and their activation. Whereas CD14+ monocytes stimulate the TGF-β1 pathway through the expression of the integrin αvβ8, this is not observed for CD16+ monocytes [110].

After exiting the circulation, blood-derived monocytes rapidly differentiate into highly phagocytic active macrophages which are innate immune cells that are abundant in tissues [111]. In many organs, including the lung, they are divided into tissue-resident macrophages (TRMs) and “monocyte-derived” macrophages (Mo-Ms) [38, 82, 111]. While the latter are continuously generated and replaced from the BM hematopoietic system, TRMs are derived during embryonic development from erythro-myeloid progenitors of the yolk sac (YS) and fetal liver monocytes [112, 113]. TRMs are abundant in lung alveoli and contribute to the formation of the alveolar niche in the first week of life [114]. TRMs can develop from fetal monocytes by gaining an established phenotype shortly after birth in response to instructive cytokines. Furthermore, they are able to self-maintain throughout life [115]. YS-derived macrophages and fetal monocytes can arise as identical alveolar macrophages, while mostly the fetal monocytes colonize the alveolar area [116]. Tissue-resident alveolar macrophages (TRM-AM) play a central role in lung development, tissue homeostasis, and immune responses. Their absence leads to infections and alveolar proteinosis due to loss of protein clearance [115]. Their contribution to the formation of the alveolar niche in the first weeks of life as well as to disease development is of current interest [112–115, 117, 118]. Although the exact contribution of the macrophage lung in the pathogenesis of BPD has not been elucidated yet, adequate early macrophage activation of macrophages is shown to be crucial to protecting infants lungs from the development of BPD [119].

Several studies have shown that Mo-AMs play an important role in endotoxin-induced, acute lung inflammation [52, 120–122]. However, as the maturation of the lung macrophages is a postnatal process, preterm and term newborns are rendered more susceptible to disease until a “catch up” is achieved if at all [123], further aggravated by the reduced number of lung TRM-AM [124]. Monocytes and Mo-AMs from preterm infants less than 30 weeks of gestation, even show impaired pro-inflammatory cytokine production, i.e., IL-8, IL-1β, IL-6, and TNFα [9, 125–127], together with a deficient pathogen response in comparison to adults, enhancing the infection and damage susceptibility further [128].

Both MV and oxygen exposure of the underdeveloped lung provokes oxidative stress [63, 129], in turn leading to increased expression of pro-inflammatory cytokines such as TNFα, IL-8, IL-1β, and IL-6 tracheobronchial aspirates [130–132]. These multiple-hit events are self-perpetuating when adding the crosstalk with the epithelial cells engaged in cytokine expression, NF-κB activity, and ROS production which results in monocyte recruitment and macrophage differentiation [133].

### Disease modeling of BPD by focusing on innate immune cells

The role of neutrophils and monocytes in BPD onset and progression is studied using different methods and models, mainly relying on 2D cell cultures and measurement in clinical samples to complex animal models (Table 1). Several studies addressed the innate immune system in the lung compartment by the use of BAL, TA specimens, cord blood cells, and serum from preterm and term neonates in the first week of life [24, 45, 134–136]. Patient-derived samples are valuable pre-clinical tools for defining predictive biomarkers in disease development. However, isolating the untouched innate immune cells for in vitro studies can significantly affect their phenotype [137]. Several non-human species studies, which used mice, sheep, and baboons have been performed to study the BPD [13, 71, 124]. The experimental use of both prenatal and postnatal lung injury including MV and oxygen exposure in wild-type and transgenic animals attempts to investigate the cellular and molecular mechanisms of the development of disease in a preclinical setting [8, 13, 124, 138]. Differences between animal and human development of immune functions render these studies challenging. In addition, different in vitro models using primary cells or primary cell lines are added to gain insight into injury-relevant mechanisms of the innate immune cell [49, 139]. However, to the best of our knowledge, realistic in vitro models which mimic the physiological and pathophysiological of the lung with a focus on the migration of neutrophils and monocytes under static or dynamic conditions have not been employed in the field of BPD research thus far [139–141].

*To provide an overview, we summarized relevant models for studying neutrophil and monocyte functions in the injured developing lung in chronological order in* Table 1.

**Table 1:** Selected research on the role of neutrophils and monocytes in BPD

Year	Model/specimen	Main findings on the role of neutrophils and monocytes in BPD	Ref
**1984**	Human neonatal BAL	Neutrophil influx and imbalance between elastase and alpha 1-proteinase inhibitor contribute to BPD development.	[134]
**2001**	Human neonatal TA	MCP-1 and IL-8 increase describes BPD and is correlated to oxygen exposure and duration of MV.	[135]
**2003**	Fetal and neonatal lamb	Monocytes from preterm and term lambs differ from the adult cells regarding inflammation initiation and resolution.	[124]
**2004**	Human neonatal cells	Decreased CD18 expression on neutrophils and monocytes and CD62L on neutrophils are early predictors of BPD.	[76]
**2004**	Neonatal rat	The combination of hyperoxia exposure and neutrophil accumulation has a pivotal role in the development of BPD.	[67]
**2007**	Human placenta and cord blood	Neonatal monocytic IL-10 production is below the needed for inhibition of release of IL-8. Suggesting exogenous IL-10 as a BPD treatment strategy.	[136]
**2008**	Human neonatal blood	Low neutrophil counts in the systemic circulation might predict BPD severity.	[142]
**2009**	Neonatal sheep	Prenatal inflammation affects fetal immune responses including the maturation of monocytes to AMs.	[13]
**2011**	Neonatal mice	MV-O_2_ leads to an increased accumulation of neutrophils and monocytes/macrophages in the lung.	[143]
**2013**	Neonatal mice	Perinatal inflammation and postnatal hyperoxia mark the activation of the macrophages which can be enhanced by IL-1Ra.	[144]
**2015**	Neonatal mice	Increased TGFβ1 expression in leads to apoptosis and monocyte and macrophage infiltration.	[145]
**2016**	Neonatal mice	MV increases the infiltrating monocytes and cytokine expression in the lungs of TNFα^-/-^ mice in comparison to the WT.	[138]
**2018**	Neonatal mice	Csf1r expressing monocyte/macrophage lineage are critical mediators of arrested alveolarization.	[83]
**2019**	Human neonatal blood	The elevated neutrophil-to-lymphocyte ratio is an early predictor of BPD.	[12]
**2019**	Neonatal mice and human neonatal TA	The presence of neutrophil-derived pathogenic in BPD lung secretion promotes extracellular matrix destruction.	[66]
**2019**	Human neonatal TA	Association between early changes in monocyte-specific IL-1 cytokine and evolving BPD.	[24]
**2019**	Neonatal mice and human neonatal TA	Inhibition of miR199a-5p improves lung vascular leak and decreased BALF total cell counts including macrophages and neutrophil influx.	[146]
**2020**	Neonatal rat	Long-term hyperoxia exposure reduced the number of peripheral blood neutrophils in BPD.	[77]
**2020**	Human neonatal TA	Identify higher expression of inflammatory mediator genes on the first day of life as a predictive BPD signature.	[123]
**2021**	Neonatal rat	Upregulated monocyte and neutrophil chemotaxis genes and involvement of the pulmonary T cell receptor signaling pathway in BPD.	[147]

## Conclusions and outlook

In summary, the immature lung undergoing injury is characterized by increased concentrations of proinflammatory cytokines as exemplified by measurements in TA obtained from very premature infants in the first days of life. While infections, oxygen exposure, and MV contribute to BPD development, the affected innate immune functions play a critical role in this complex disease of prematurity. During the past few years, significant efforts have been made to examine the role of inflammation-induced lung injury considering both prenatal and postnatal conditions while using human samples and animal models. Studies showed that a preterm neonate with a structurally and functionally immature lung, paired with an underdeveloped immune response exhibit an increased pro-inflammatory response when BPD develops. Therefore, targeting the inflammatory process is considered a therapeutic or preventive approach for BPD.

Nonetheless, many conflicting results remain. Prematurity as such results in a smaller pool of neutrophils and monocyte/macrophages and a subsequently lower capacity to secrete cytokines and reach pathogen clearance. In BPD, however, elevated numbers of neutrophils and monocytes, and their inflammatory cytokines can be detected in the diseased lung. The differences in local and systemic concentrations may relate to mechanisms driving tissue accumulation despite reduced BM production rates. Targeting the number of peripheral blood immune cells has been suggested as a therapeutic strategy in BPD. Nevertheless, this approach neglects the developmental functions, the same innate immune cells, and their signaling molecules hold.

Concerning cell-specific effects and their interplay, neutrophil presence and activation marking the first line of defense hold a crucial function in the BPD lung and correlate with the severity of symptoms. Detection and characterization of a pronounced monocyte-centered inflammatory response in preterm infants with later BPD mark the “second wave” of events. However, the challenging question is how physiologic defense signaling is changed into an immune reaction while infection clearing and termination of inflammation are not reached. The subsequent release of damage signals initiates the detrimental cascade of alveolar and airway injury.

Future studies need to overcome current limitations that exist due to a variety of reasons. First of all—given the nature of the patient collective—only a limited number of preterm infants can be enrolled in the majority of studies. Secondly, volume restrictions of clinical samples obtained from preterm infants limit the experimental settings in addition to the non-physiological activation of primary cells during isolation and culture. Hence, reliable analysis techniques and the avoidance of solely targeted approaches need to be combined with optimized “preclinical” in vitro models to overcome the described challenges. The establishment of large datasets standardized or harmonized between centers and groups including single-cell analysis will help to provide valuable resources for studies to come regarding both developmental and injury functions of the innate immune system. Preclinical animal models—despite the differences in immune regulation processes and marker expression when comparing mice and men—should be combined to tackle context-related knowledge gaps putting in vitro findings into perspective concerning cellular crosstalk and spatial resolution.

In the future, our understanding of the role of the immune system in BPD will become increasingly important as more sophisticated therapeutic and diagnostic possibilities emerge that may allow targeted approaches to treat and monitor disease-relevant processes in a population tackling critical steps of organ development.

## Data Availability

Not applicable.

## Abbreviations

MV: Mechanical ventilation
GA: Gestational age
BAL: Bronchoalveolar lavage
TA: Tracheal aspirate
bpd: Bronchopulmonary dysplasia
nCLD: Neonatal chronic lung disease
AMs: Alveolar macrophages
TRMs: Tissue-resident macrophages
Mo-Ms: Monocyte-derived” macrophages
TR-AMs: Tissue-resident alveolar macrophages
Mo-AMs: Monocyte-derived alveolar macrophages
RDS: Respiratory distress syndrome
BLF: Bronchoalveolar lavage fluid
NETs: Neutrophil extracellular traps
TGF-β1: Transforming growth factor
G-CSF: Granulocyte colony-stimulating factor
ROS: Reactive oxygen species
MCP: Monocyte chemoattractant proteins
MIP: Macrophage inflammatory proteins
ECM: Extracellular matrix
SERPIN-B1: Serine protease inhibitor
NE: Neutrophil elastase
MMP-9: Metalloproteinases-9
YS: Yolk sac
